# A Comprehensive Review on the Fatigue of Wood and Wood-Based Materials

**DOI:** 10.3390/ma18225118

**Published:** 2025-11-11

**Authors:** Gregor Gaberšček Tuta, Gorazd Fajdiga

**Affiliations:** Biotechnical Faculty, University of Ljubljana, Jamnikarjeva 101, 1000 Ljubljana, Slovenia; gorazd.fajdiga@bf.uni-lj.si

**Keywords:** wood, fatigue, physical properties, mechanical properties, measurement techniques, prediction models, simulation, future research

## Abstract

The fatigue of wood is becoming increasingly important in modern engineering, as the safety of the structure must be guaranteed and the use of materials must be optimized at the same time. Predicting the fatigue behavior of wood remains a challenge for many researchers. Interest and the number of studies in this field have increased, highlighting the need for a comprehensive overview of the current state of knowledge on wood fatigue. In this paper, we focus on the study of the fatigue of wood-based materials to understand the similarities and peculiarities of fatigue behavior compared to other engineering materials and to identify opportunities for new research. We present the influence of physical and mechanical properties on fatigue life and identify similarities in the fatigue behavior of wood, polymeric materials and steel. The basic properties that differentiate the fatigue life of wood from that of other materials are heterogeneity, orthotropy, viscoelasticity, hygroscopicity, mechanosorptivity and the lack of a clear threshold value for fatigue strength. The differences in fatigue life between solid wood and laminated wood are not uniformly defined by researchers. We provide an overview of the measurement methods used to monitor the fatigue state, the models used to predict fatigue life and the simulations of the stress–strain response to cyclic loading. We identify areas where wood is subject to fatigue and determine which areas are most critical under cyclic loading. We make suggestions for further research that would contribute significantly to a better understanding and management of wood fatigue. Due to the wide variety of wood species used in the studies, it is impossible to compare the results. In order to obtain a comprehensive overview of the response of wood to fatigue under different test conditions, the test methods need to be standardized.

## 1. Introduction

The fatigue of wood has been studied for at least 70 years. Trends indicate that interest has increased in recent years due to greater awareness of environmental issues. The high stiffness-to-weight ratio and the ability to store atmospheric carbon give wood a significant advantage when choosing between alternative materials such as steel, concrete, and polymers. Wood is a hygroscopic, heterogeneous, and orthotropic material, so its response to mechanical loads is difficult to predict. This unpredictability results in higher material consumption. Wood is a renewable resource that must be used sustainably. Therefore, understanding its response to monotonic and cyclic loads is essential for optimising the use of raw materials.

Earthquakes, hurricanes and storms are expected to occur more frequently in the environment. Such events can severely damage newly constructed structures if they are not designed to withstand fatigue. Wood has many positive characteristics, such as aesthetics, ease of fabrication, light weight and environmental friendliness. Because of these properties, it has long been used for simple applications. For more demanding applications, where load-bearing capacity, dynamic behaviour and safety are critical, it is necessary to understand its lesser-known properties and influencing factors. The fatigue strength of wood has been studied for a long time, but remains largely unexplained. It was once believed that wood was not susceptible to fatigue and had no fatigue threshold, which led to its use as a structural material alongside steel [1,2]. During the Second World War, aircraft were built from wood, in which no fatigue occurred. Fatigue was mitigated by safety from creep. For this reason, there was no need to research the fatigue of wood [1,3,4].

It has long been known that prolonged loading leads to creep [1], a form of fatigue. Depending on the magnitude of the static load, there is a specific service life after which failure occurs [5]. This was the first method used to determine the fatigue life of wood under cyclic loading, as the duration of loading cycles was accumulated. Later, it became clear that such a simplification was not optimal. Wood does not exhibit distinct regions on the Wöhler curve (S-N curve) as steel does, so the threshold value for fatigue strength is unclear [6]. However, it is recognised that wood has a fatigue limit [1]. Much is now known about how wood and wood composites behave under fatigue, but this knowledge does not always translate from specimens to actual products. Furthermore, due to anisotropy and heterogeneity, it is largely not transferable between wood species or even within a single species. Ultimately, wood is a material created by nature, not by humans.

The first generation of Eurocode 5 standards includes an annex on the fatigue of timber, which is limited to a small number of timber structures. Fatigue-based design is not included in the standard, as there is not yet a reliable analytical model for predicting the fatigue life of timber structures [7,8]. Fatigue is only addressed in the standard through factors that reduce the allowable stresses when structures are subjected to vibrations. A new generation of Eurocode 5 standards is being prepared, in which the effects of fatigue on timber structures will hopefully be defined more precisely. The effects of the environment and the physical properties of individual wood species on the response to cyclic loads must be studied. The description of the mechanical properties of wood is much more complex than that of steel. Wood properties are described by three moduli of elasticity, three shear moduli, and six Poisson’s ratios [9]. Despite efforts to determine the mechanical properties more precisely, discrepancies remain between the values obtained by different methods for the same wood sample [10,11]. Even greater discrepancies exist in the models used to predict the fatigue life of wood-based materials. Some argue that wood behaves more like steel, where the S-N curve is defined by the number of cycles. Others claim that the fatigue of wood is better described by the DOL (Duration of Load) model, which is defined by the time under load [12]. Various models have emerged due to the numerous influencing factors. For example, temperature significantly affects the fatigue life of steel, while humidity is a similarly influential factor for wood. In addition, the frequency, the dynamic load factor R (explained in Section 3.1), and the shape of the load cycle have a significant influence on fatigue life [1,6].

The variability of the mechanical properties of wood is lower in laminated products such as glued laminated timber (glulam), plywood, laminated veneer lumber (LVL) and similar products that combine wood lamellas with physical and mechanical properties that are as similar as possible. The result is wooden elements of any dimensions with a higher degree of homogeneity.

Few researchers are familiar with wood fatigue, as it remains a highly specialised research area. The problem is that allowable design stresses are based on static strength, even though fatigue accounts for 90% of material failures. As a result, structural elements are oversized to compensate for the unknown factors related to fatigue. To preserve wood as a renewable resource amid increasing demand for green and sustainable products, it is essential to optimise wood-based structures and products. Therefore, wood fatigue is an important research area that should be addressed by more researchers. To facilitate this, the research community must first be introduced to the field through a comprehensive review of the existing literature. In this study, we focused on the cyclic fatigue of wood and conducted an extensive literature review. In Section 2, we examine how wood-specific physical properties influence fatigue life under cyclic loading. Combinations of individual effects can have either positive or negative impacts on fatigue life. In Section 3, we describe how individual properties and their combinations affect fatigue life under cyclic loading. Wood is used in various forms, from multi-storey buildings to furniture. In Section 4, we investigated the dynamic properties of the most common wood species and wood composites. The properties of wood-based materials and their response to fatigue are assessed using various measurement methods. In Section 5, we examine non-destructive measurement methods for determining physical and mechanical properties and for monitoring the response to mechanical cyclic loading. We also reviewed models for predicting service life and simulations of the stress–strain response under cyclic loading. Examples of cyclic loading of wood are presented in Section 6. Finally, in Section 7, we provide suggestions for further research that would significantly contribute to a better understanding and management of wood fatigue.

## 2. Influence of Physical Properties

### 2.1. Density

Density is an important physical indicator, as many mechanical properties are related to it. It is closely linked to moisture content. The density of cell walls is higher than that of water. However, due to the porous structure of wood, the overall density is lower than that of water. Because of this porosity, density can change considerably above the fibre saturation point (FSP). Density changes up to the FSP due to the amount of bound water; then, as water begins to fill the cell lumina, density increases rapidly. It is important to compare density at the same moisture content or when the wood is absolutely dry. The strength and, consequently, the fatigue life of wood depend on its density. It is therefore important to compare properties under fatigue at the same moisture content and density of the samples. In addition to moisture content, growth conditions affect density. The faster a tree grows, the lower the wood density. This is because there is more space between growth rings, resulting in more early wood (low density) and less late wood (high density). Density varies across the cross-section due to tension wood, compression wood, and knots. All these factors contribute to the variability of density and, consequently, to different fatigue lives of samples within the same wood species.

To date, extensive research has been conducted in the field of wood fatigue. Many studies have examined various factors affecting the fatigue life of wood. Miyoshi et al. [13] investigated the influence of density on the modulus of elasticity, strength, and failure strain, finding that the relationship is linear, but only in certain load directions relative to the grain direction. Earlier studies confirm the expected improvement in mechanical properties at higher densities [1]. In these studies, displacement control is used, where the displacement remains constant but the load decreases due to the reduction in the elastic modulus [4]. In recent research, where tests are performed under constant load, it has been found that the dynamic properties are better at higher densities [14]. However, in Klemenc’s study, spruce samples with higher density [15] were found to have lower fatigue life. This may be due to the use of a statistical model with a small sample size. Overall, researchers agree that higher density wood has better fatigue resistance [3,16].

### 2.2. Moisture

The moisture content is determined by the ratio of the mass of water in the wood to the mass of absolutely dry wood. Most wood used in construction and products attains a moisture content between 8% and 20%, which constantly changes depending on the temperature and relative humidity of the environment. The amount of free water does not significantly influence the mechanical properties; rather, it is the amount of bound water that has a greater effect. For most wood species, the fibre saturation point (FSP) is between 25% and 30% moisture content. From absolutely dry wood to the FSP, the mechanical properties change considerably. The modulus of elasticity, shear modulus, and both static and dynamic strength also vary with moisture content. The higher the moisture content, the lower the static strength; this relationship is linear, as Wang et al. confirmed for six wood species [17]. Majano-Majano et al. found that the toughness of beech and birch increases with increasing moisture content [9]. Mechanical properties decrease with higher moisture content, except for toughness. Fatigue life also decreases as moisture content increases, as shown in the literature [4,18,19,20,21,22]. For example, Tsai et al. tested the fatigue life of LVL at a dynamic load factor R = 0.1 and three different moisture content levels. The difference in fatigue life at 80% of flexural strength between samples with 35% and 5% moisture content was significant at $0.8\cdot 10^{6}$ cycles [4].

The moisture-dependent mechanical properties also depend on temperature, as the relative humidity of the environment changes with temperature. Fatigue at higher frequencies, as used in experiments to shorten test times, leads to an increase in temperature at points of maximum deformation. Whether such a temperature increase affects drying was investigated by Kommers et al., who found that a 10 °C increase in temperature reduced moisture content by 1% [23]. It is important to note that they tested in displacement control mode, so their results would need to be verified by a similar study using load control. Clorius et al. tested with the constant load method at a frequency of 10 Hz, but could not observe any drying due to the increased temperature [18]. The drying effect is not consistent with studies using high-frequency loading. Wood whose moisture content has changed significantly within a short period exhibits internal stresses [24], which can significantly alter the response to mechanical loads. When moisture is exchanged with the environment, the wood shrinks and swells, allowing damage accumulation, which can lead to fatigue damage [25].

### 2.3. Temperature

**Figure 1:**
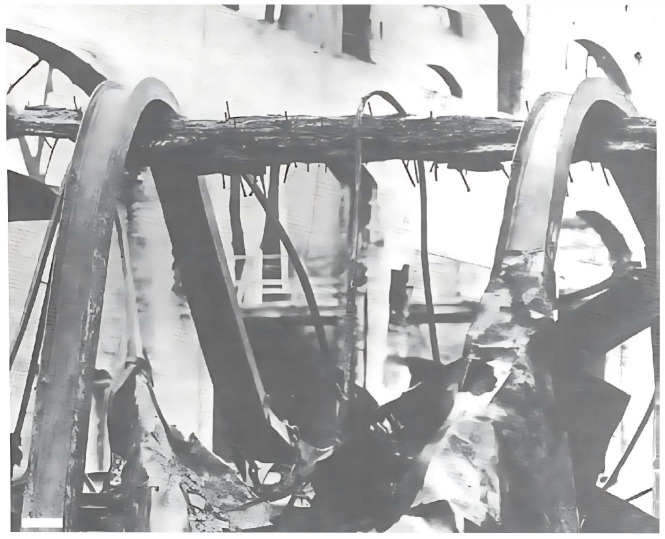
Steel members yielded by the heat are supported by a charred wood beam [31].

Temperature directly affects the mechanical properties of wood. Gerhards reviewed research up to 1980, showing that the modulus of elasticity, shear modulus, tensile strength, and compressive strength all decrease as temperature increases [26]. More recent studies confirm this trend [27,28,29]. However, wood as a building material is more resistant to high temperatures than steel. For example, in a fire, steel beams lose strength at high temperatures and deform under their own weight. In contrast, wood chars on the surface but retains a solid core, preventing collapse during a fire [30], as shown in Figure 1.

Temperature can have a considerable indirect influence on the mechanical properties of wood. Relative humidity changes with temperature (Section 2.2). Temperature has been found to have a greater influence on mechanical properties at higher moisture content [29]. Tsai et al. note that there are no data on how temperature affects fatigue or what influence it would have on the S-N curve [4]. Salmen tested the degree of damage at temperatures from 80 °C to 140 °C under cyclic loading at different frequencies. He found accelerated damage formation at higher temperatures [32], indicating that at least at certain load frequencies, fatigue life is negatively affected by increased temperature. Cyclic loading is not necessarily only mechanical, but can also be thermal. Thermal cyclic loading causes shrinkage and expansion, leading to material fatigue. At constant moisture content, the thermal expansion coefficient of wood is between 3.1 and 4.5 × 10^−6^ K^−1^, in the longitudinal direction, approximately 31 × 10^−6^ K^−1^, in the radial direction, and approximately 40 × 10^−6^ K^−1^ in the tangential direction [33]. For comparison, steel has a coefficient of thermal expansion of around 10 × 10^−6^ K^−1^, which makes wood more susceptible to thermo-mechanical fatigue in the radial and tangential directions. Shrinkage and expansion are not solely due to temperature, but depend largely on moisture content, which in turn is a function of temperature. During shrinkage and expansion, microcracks can form in the cell walls [34].

### 2.4. Fiber Orientation Relative to Load Direction

**Figure 2:**
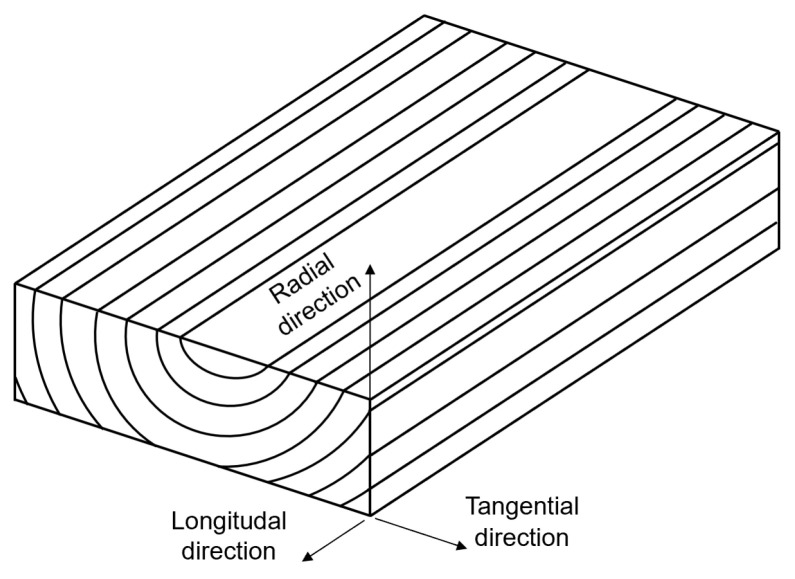
Definition of anatomical sections in wood.

Wood has three anatomical directions, longitudinal, radial, and tangential, which result from the tree’s growth, as shown in Figure 2. The mechanical properties depend on the direction of the mechanical load relative to the anatomical direction.

The modulus of elasticity is highest in the longitudinal direction, lower in the radial direction, and lowest in the tangential direction. The static load-bearing capacity follows this pattern: it is highest in the growth direction, lower in the radial direction, and lowest in the tangential direction. The load-bearing capacity in each anatomical direction depends on the type of mechanical load. An element loaded in tension in the longitudinal direction has a higher load-bearing capacity than one loaded in compression. This also applies to loading perpendicular to the fibres [35].

**Figure 3:**
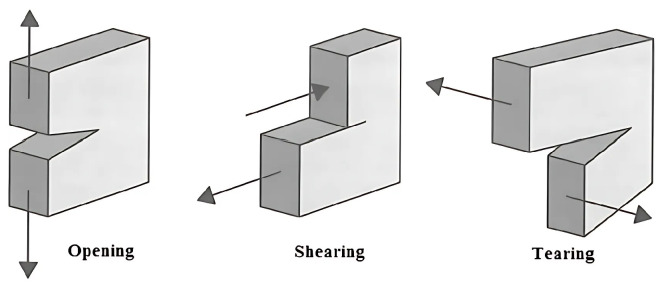
Three modes of fracture [36]. Arrows indicate the direction of acting mechanical load.

The results of loading tests in different anatomical directions can be compared, but only when using one type of loading method. In general, the properties under static loading are similar to those under cyclic loading. The fracture mechanisms can be divided into three typical modes, as shown in Figure 3. Mode I is a tensile load perpendicular to the plane (opening), Mode II is an in-plane shear load (shearing), and Mode III is an out-of-plane shear load (tearing). In practice, a combination of two or more fracture modes often occurs.

#### 2.4.1. Tensile Load Parallel to the Grain

**Figure 4:**
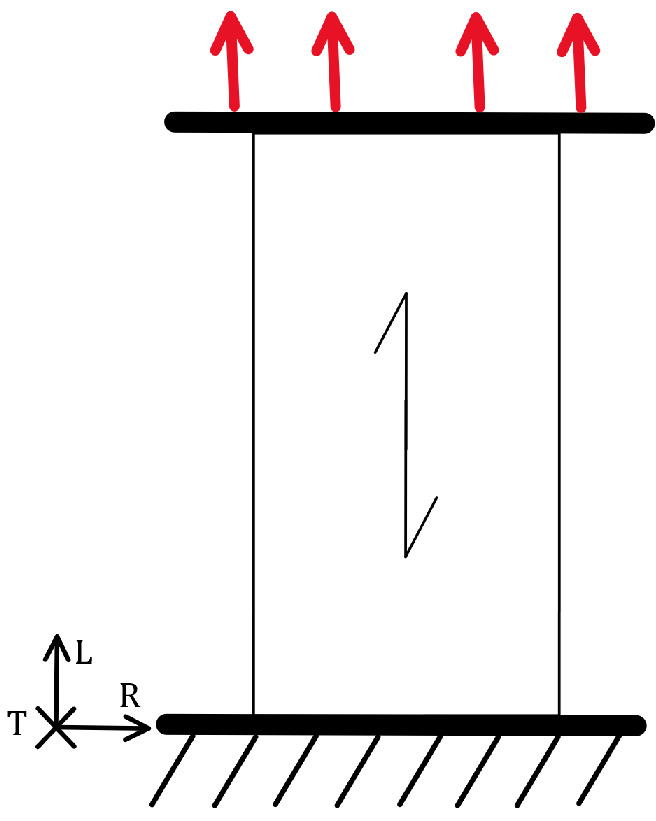
Tension load parallel to the grain. Arrows indicate the direction of the applied mechanical load.

In the longitudinal direction under tensile load, as shown in Figure 4, both the static load capacity and stiffness are at their highest. The same applies to fatigue strength, which is also greatest under this type of load [37]. The orientation of the wood structure relative to the load ensures that the load acts along the elongated wood cells, which can withstand the highest tensile stress.

**Figure 5:**
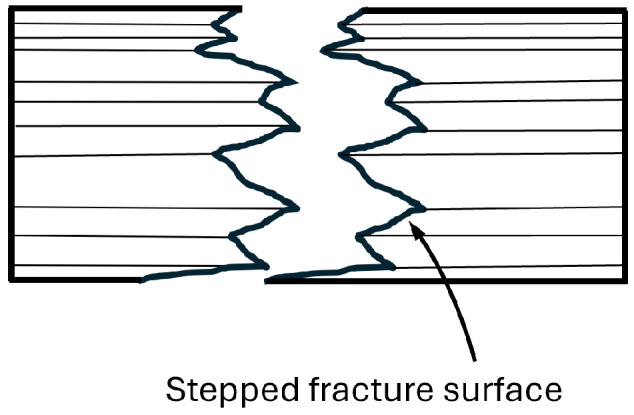
Typical failure of wood in tension parallel to the grain.

Such a load is rarely used in practice, as indicated by the limited literature. Even less is known about the fatigue response under these loading conditions. It is known that fatigue strength in tension parallel to the grain is slightly higher than in shear, as reported by Kretschmann, who observed approximately 30 × 10^6^ cycles at *R* = 0.1, a frequency of 15 Hz, and a load representing 50% of the static load capacity [38]. From a macroscopic perspective, the fracture mechanism begins with cracks that grow to a critical size over subsequent cycles. Numerous longitudinal cracks lead to short transverse cracks. At the cellular level, areas of pulled-out cell clusters and distinct horizontal cracks are common, resulting in a stepped fracture surface [37], which can also be observed macroscopically in Figure 5 as cross-grain cracks between growth rings.

#### 2.4.2. Tensile Load Perpendicular to the Grain

**Figure 6:**
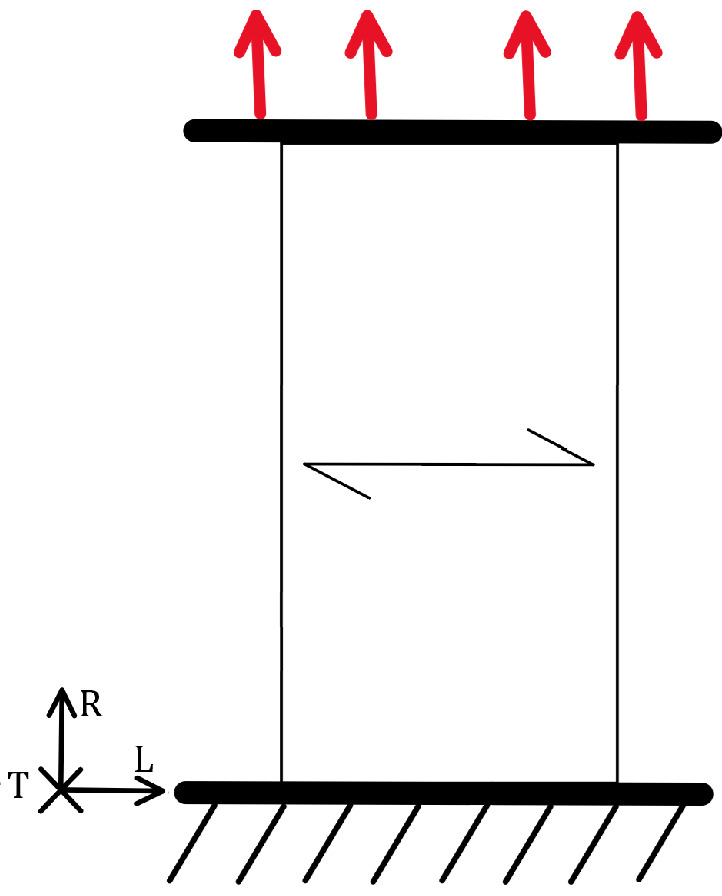
Tensile load perpendicular, radial to the grain. Arrows indicate the direction of the applied mechanical load.

**Figure 7:**
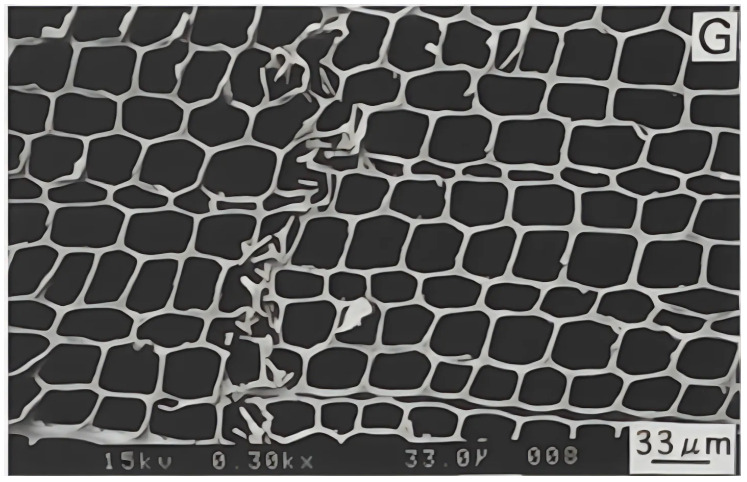
First break on compressive load perpendicular to the grain [41]. SEM image at 15 kV accelerating voltage, 0.30 kx magnification, and 33.0 mm working distance. Scale bar = 33 μm.

**Figure 8:**
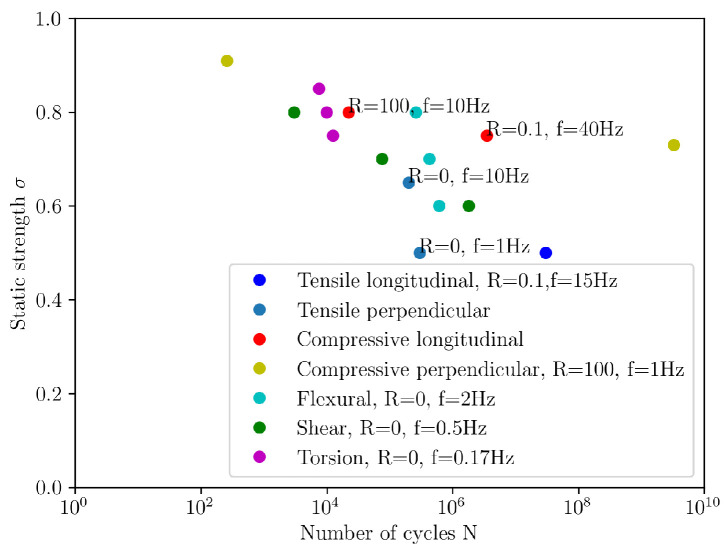
Results of fatigue life in different load directions and various testing conditions [3,12,18,38,39,42,43,44].

Perpendicular loading includes both the radial and tangential directions. Figure 6 shows the tensile load in the radial direction. The softwood tracheid structure, shown in Figure 7, clearly illustrates why load capacity is lower in the perpendicular direction than in the longitudinal direction. The fibres in the transverse direction are arranged as parallel tubes or honeycomb structures, which deform or may even collapse if high enough load perpendicular to the grain is applied. Consequently, the static and dynamic load capacities in tension are lower in this anatomical direction; in fact, they are the lowest compared to other anatomical directions [1]. Clorius investigated this type of loading [39]. Approximately 0.2 × 10^6^ cycles are achieved at *R* = 0, a frequency of 10 Hz, and 65% of the static load capacity in the direction perpendicular to the fibres [12], or 0.3 × 10^6^ cycles at *R* = 0, a frequency of 1 Hz, and 50% of the static load capacity [39]. To provide a general overview, we have presented the results of these and other authors in a combined σ−N diagram in Figure 8, which contains the results of cyclic tests in different directions and under different conditions. The damage mechanism in this loading direction is poorly understood. Under static loading, the fracture is brittle due to the tensile load, and the fracture surface is rough and wavy [40]. Brittle fracture can also be expected under fatigue loading, but the fracture surface may be flatter, similar to the fatigue fracture of metals.

#### 2.4.3. Compressive Load Parallel to the Grain

**Figure 9:**
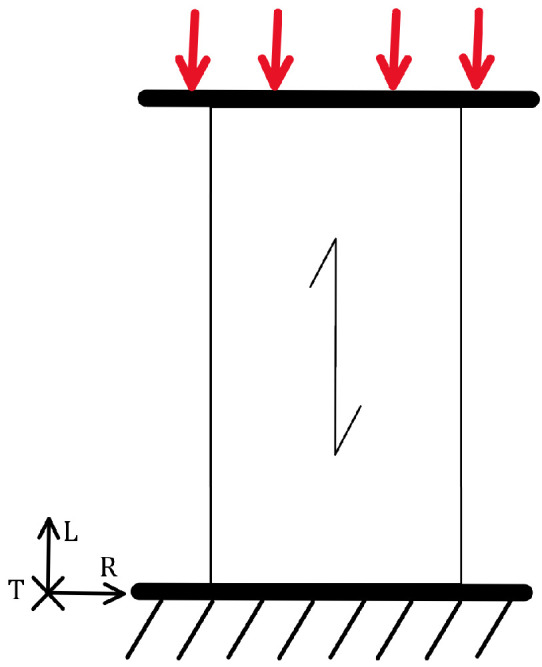
Compressive load parallel to the grain. Arrows indicate the direction of the applied mechanical load.

Columns, beams, and various connections in a structure are subject to compressive loads. Elements subjected to tensile and shear loads exhibit a characteristic quasi-brittle fracture, while those subjected to compressive loads exhibit a characteristic ductile fracture. Compressive loads in the fibre direction, as shown in Figure 9, are more critical than tensile loads. The static compressive load capacity is about half that of the tensile load. Fatigue behaviour follows that under static loading, as Schonbauer et al. have found [45].

Two examples of cyclic compressive loading in the fibre direction, which demonstrate how strongly the test conditions influence the results, were published by Clorius [18] and Kretschmann [38]. The first experiment was conducted at R=100, with a frequency of 10 Hz and a load amplitude representing 80% of the static load capacity. In this case, the samples were subjected to approximately 0.022 × 10^6^ load cycles [18]. The second experiment used a dynamic load factor that does not reflect the cyclic compressive load, *R* = 0.1. Nevertheless, the author provides results for cyclic fatigue under full compression. The loading frequency was 40 Hz, with a loading amplitude corresponding to 75% of the static loading capacity. In this case, the samples withstood approximately 3.5 × 10^6^ loading cycles [38]. The difference of several million cycles in fatigue life must be due to different frequencies and possibly different load ratios, as the difference in load amplitude is minimal. The results are shown in the diagram in Figure 8.

**Figure 10:**
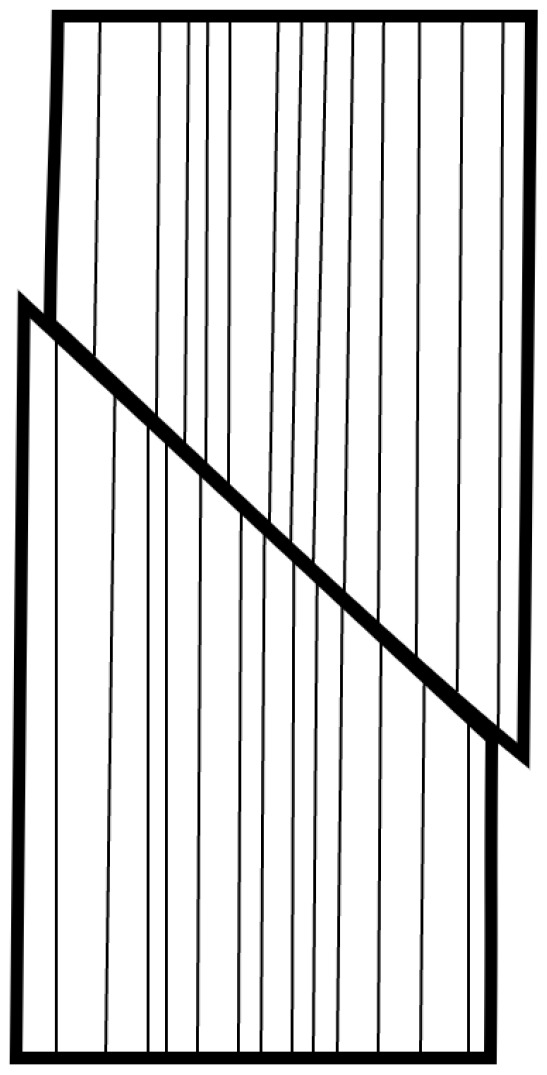
Failure mode on compressive load parallel to the grain.

The first signs of damage caused by compressive loading are crushed cell walls (tracheids/tracheae) known as kinks. Bonfield et al. [37] described the damage mechanism during fatigue and found that the cell ends are crushed at the point of failure. Macroscopically, a pronounced fracture plane can be observed at an angle of about 45° to the specimen axis, as shown in Figure 10, which results in shear stresses. In this case, the fracture surface does not form and the test is terminated when the maximum displacement is reached.

#### 2.4.4. Compressive Load Perpendicular to the Grain

**Figure 11:**
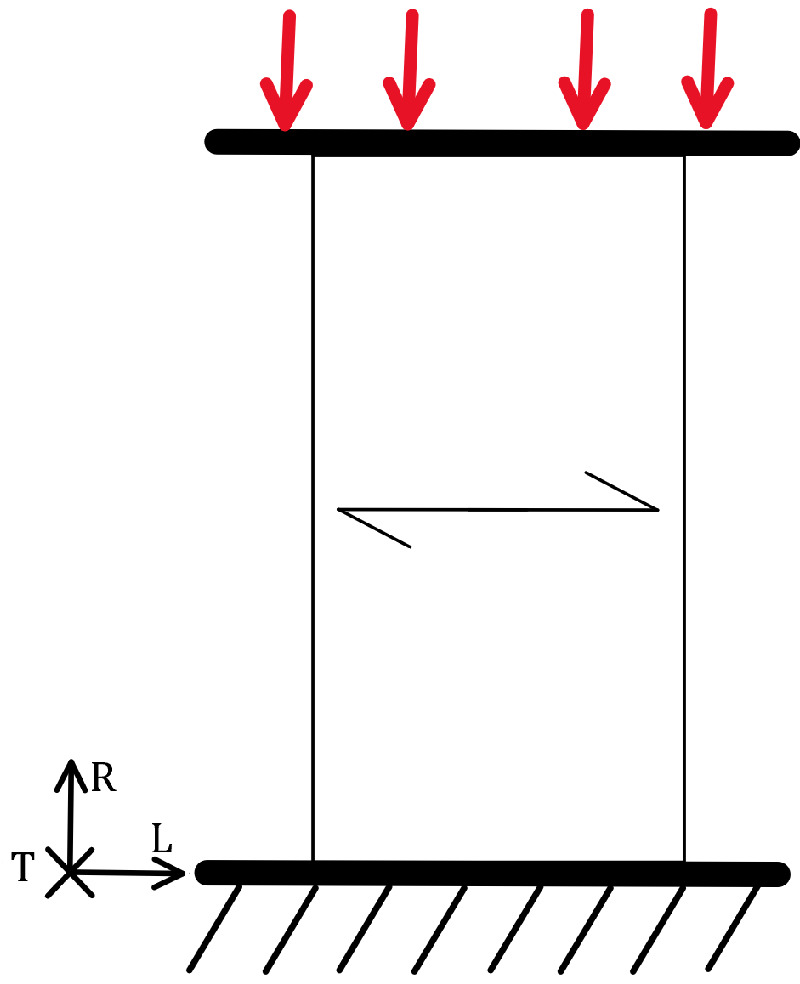
Compressive load perpendicular to the grain. Arrows indicate the direction of the applied mechanical load.

Compression loading perpendicular to the grain, as shown in Figure 11, is used in pulping for papermaking because the strength in the direction perpendicular to the fibres under compressive load is low and, consequently, less energy is required for failure [32,46].

Before pulping, the wood is fatigued at large displacements to reduce its modulus of elasticity, facilitating easier processing in the paper industry. Another area where cyclic compressive stresses occur perpendicular to the grain is in wood joints such as the Tenon Mortise or Dou Gong. These joints are particularly important for transmitting seismic vibrations, as they must provide structural strength while damping vibrations during an earthquake. To determine how these joints respond to fatigue, wood is subjected to cyclic compression perpendicular to the grain [47]. The energy dissipation is observed; the greater the dissipation, the better the structure performs during an earthquake. Ogawa et al. [42] tested Japanese cypress at R=100 perpendicular to the fibres at a frequency of 1 Hz and found that the fatigue strength is approximately 60% of $\sigma_{0.05}$ or 43% of $\sigma_{max}$. The reported fatigue strength is higher than the 15-35% of the static strength reported for softwood by others [4]. Their results are shown in the diagram in Figure 8. Ando and Onda [41] described the fracture mechanism in a monotonic test of radially compressed samples. They discovered the so-called first fracture, in which only one line of tracheids near the growth rings broke. This fracture occurred suddenly, as is typical for wood under radial compression. At the moment of the first break, the force ceases to increase while the deformation continues to increase. The force begins to increase again when the fractured cells start to compress and mash, as shown in Figure 7. From this, it can be concluded that when fatigue occurs in this direction, a similar fracture mechanism to monotonic loading would occur, with the difference that the lines of initial fractures would appear at several different locations.

#### 2.4.5. Flexure Load

**Figure 12:**
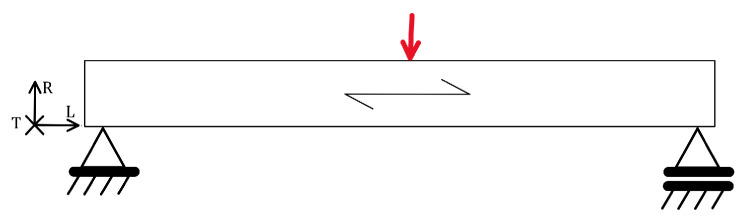
Flexure load. Arrow indicates the direction of the applied mechanical load.

The most characteristic load for wood is the flexural load, as shown in Figure 12, which is why it is also used in the characterisation or classification of sawn timber into quality classes. Just as the tensile test is used to determine the main mechanical properties of steel, the flexural test serves the same purpose for wood.

Many studies address flexural strength [48,49,50,51,52] and fatigue life under cyclic flexural loading [3,4,15,53,54] in various applications and modelling. A three-point or four-point bending load is used for testing. There is no prescribed standard for testing wood for fatigue, so the test is conducted similarly to the static test described in ISO 3133:1975 [55] or ISO 13061-4:2014 [56]. Fatigue tests with flexural loading are used to investigate the effects of density, moisture, knots and notches, loading frequency and dynamic loading factor, and to develop fatigue life prediction models. Tsai et al. found that fatigue life is largely independent of wood species when stress is normalised to static strength [4]. To compare fatigue strength between different wood species, the flexural stress is normalised to the modulus of rupture, i.e., the maximum stress just before failure. Klemenc et al. confirmed a difference in fatigue life between the tangential and radial directions for spruce wood, namely that loading in the radial direction is more damaging than in the tangential direction [15]. This is the opposite of static strength, where the tangential direction is weaker, although the sample size in their study was small, so this finding should be further validated. Yildirim et al. [3] tested pine and beech wood under pulsating flexural load at a frequency of 2 Hz and four load levels. A fatigue life of 1 million load cycles corresponded to 40% of the static strength for pine wood and 50% for beech wood. They did not define these values as fatigue strength; instead, they proposed them as allowable design stress for furniture design, which will be further discussed in Section 6.2. Their results are shown in the diagram in Figure 8, together with other loading examples.

**Figure 13:**
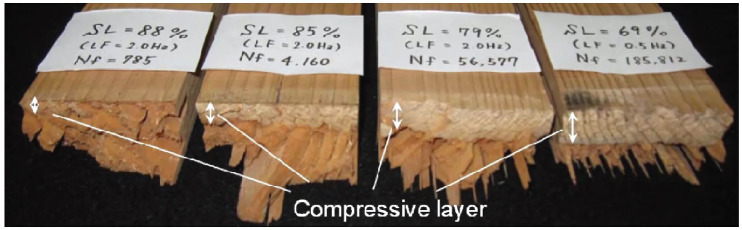
Tension and compression region in flexural load. Reproduced with permission from [54], Holzforschung; published by De Gruyter, 2014.

**Figure 14:**
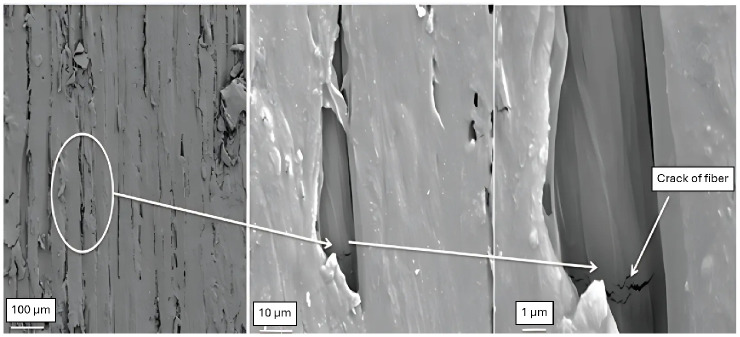
Perpendicular crack in wood cells [3].

The fracture mechanism under flexural load involves the formation of longitudinal cracks on the tensile side, which propagate until they produce long, thin, sharp splinters, while crushed areas develop on the compression side. The crushed material on the compression side causes the upper layers to lose load-bearing capacity, reducing the load-bearing cross-section. This reduction on the compression side shifts the neutral axis towards the tension side. Failure occurs when the load-bearing cross-section is reduced to a critical size. An example of failure is shown in Figure 13, where the low-cycle failure on the left displays a minimal compression layer, while the high-cycle example on the right shows a compressive layer spanning almost the entire section. Under alternating load, the tips of the splinters on the tensile side are blunter. Figure 14 shows a SEM image of the initiation of a single crack in a fibre. Therefore, we can conclude that fatigue damage in wood begins at the microscopic level.

#### 2.4.6. Shear Load

**Figure 15:**
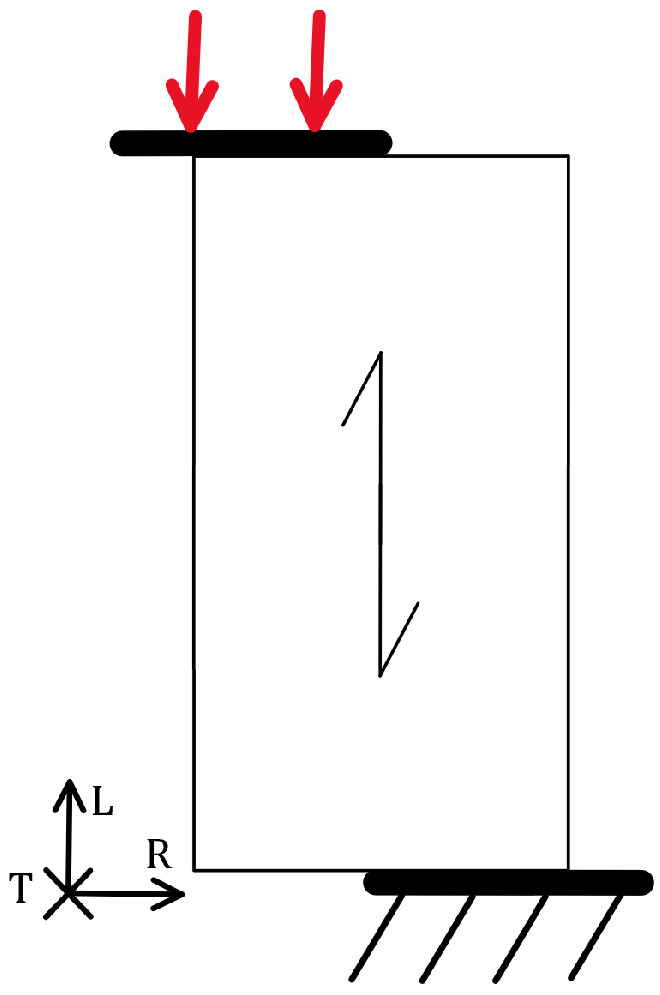
Shear load. Arrows indicate the direction of the applied mechanical load.

Wooden structural elements exposed to shear stress are mainly joints with dowels, short beams with a high cross-section [40], glued laminated timber panels, and load-bearing walls. Shear stress is analysed in three planes: the radial-longitudinal (RL) plane and the tangential-longitudinal (TL) plane, which are most common for beams in load-bearing structures, and the radial-tangential plane, which is common for joints. An example of a shear load in the TL plane is shown in Figure 15.

**Figure 16:**
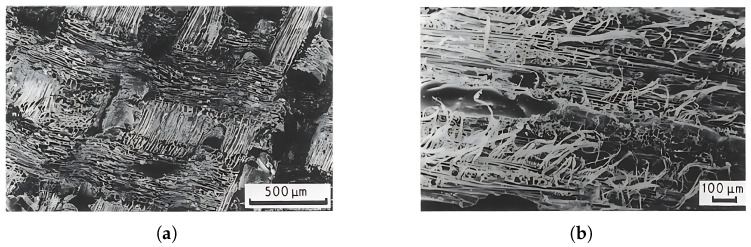
Fracture surface in shear load [37]. (**a**) Radially longitudinal plane. (**b**) Tangential longitudinal plane.

The shear strength is lower than the compressive and tensile strength [53], as is typical for polymer laminates. In wood, cellulose acts as reinforcement and lignin as the matrix [57]. In a natural lamellar composite such as wood, the shear stress in the RL and TL planes is largely transmitted only by lignin, which has lower strength than cellulose. The shear strength is therefore lower. Despite the lower static load capacity, the fatigue strength is comparable to other loading directions, as shown by the results of Sugimoto [43], which are presented in the diagram in Figure 8 among other forms of cyclic loading. According to the normalised mean stress, the fatigue strength in shear is comparable to the fatigue strength under tensile stress perpendicular to the grain and the fatigue strength under compressive stress parallel to the grain [12,18,43]. The work of Bonfield et al. demonstrates that the damage mechanism is a sudden event in which abrasion damage occurs on the fracture surfaces during fracture [37], as shown in Figure 16 for laminated veneer lumber (veneer glued in the TL plane) fractures in shear. Figure 16a shows longitudinal fibres running from top to bottom. Careful inspection of the fractured plane reveals debris left behind by ruptured radial cells. The fracture surface of the TL plane is shown in Figure 16b and displays sheared longitudinal cells that have left a fibrous surface. Most samples sheared in the wood rather than in the glue, indicating that the glued bond was stronger under fatigue loading than the wood itself.

#### 2.4.7. Torsion Load

**Figure 17:**
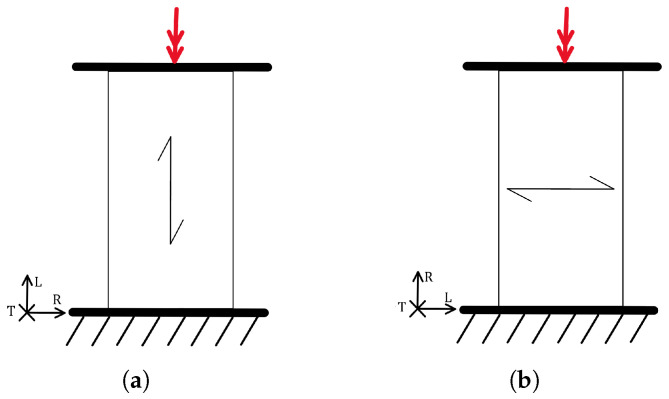
Torsion load. Arrows indicate the direction of the applied moment according to the right-hand rule. (**a**) Parallel to the grain; (**b**) perpendicular to the grain.

There are few elements in timber structures that are directly subjected to torsion; however, torsion often occurs indirectly alongside flexural loads. For example, in deep beams, torsion arises in addition to bending. Therefore, it is necessary to check both the static and dynamic torsional strength to construct more efficient and safer structures. The torsional load parallel and perpendicular to the grain is shown in Figure 17.

**Figure 18:**
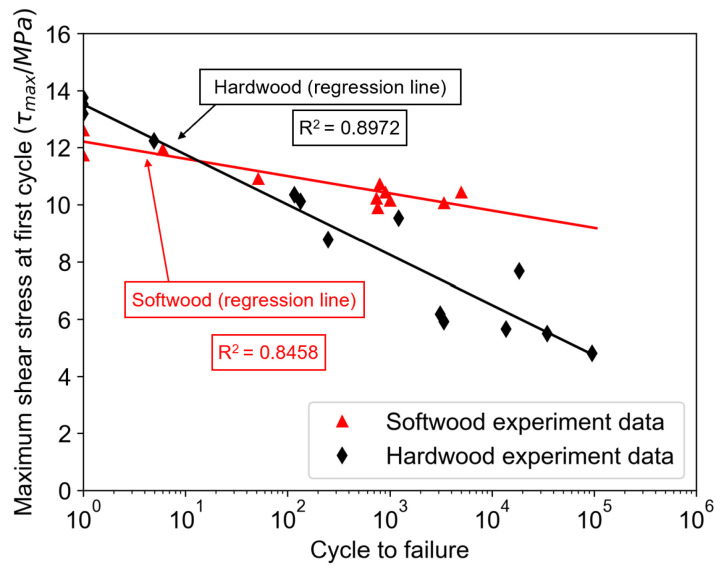
Fatigue life of softwood and hardwood [44].

The torsional strength along the grain, when the axis of rotation is parallel to the grain, is much higher than in the perpendicular direction. There is also a difference in torsional strength perpendicular to the grain between the RL and TL planes. In the RL plane, torsional strength is higher than in the TL plane, as observed in hardwood. No such clear difference is seen in softwood. In hardwood, torsional strength along the fibres is higher, and fatigue strength is better at higher load amplitudes, while in softwood, fatigue strength is higher at lower amplitudes, as shown in the diagram in Figure 18.

**Figure 19:**
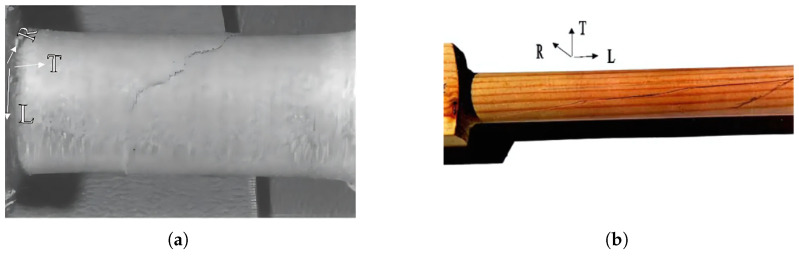
Failure in torsion load [44]. (**a**) RL plane perpendicular to torsion axis with hardwood. (**b**) Torsion axis parallel to the grain.

The results of hardwood fatigue indicate that damage occurs progressively rather than suddenly. Complete failure does not occur. Towards the end of the fatigue process, stiffness decreases and the energy loss per cycle remains almost constant until failure. Softwood behaves differently during fatigue. The energy loss per cycle decreases, and stiffness remains constant until failure, meaning that failure occurs suddenly. The diagram in Figure 8 shows the torsional fatigue strength for softwood with other loading methods and directions. It is most comparable to cyclic shear loading. The mechanism of damage formation depends on the direction of the grain relative to the loading axis. In most cases, cracks form parallel to the grain, as shown in Figure 19. In any case, the load-bearing capacity decreases significantly after the first cracks appear, less so in hardwood than in softwood [44].

In Figure 8, all referenced load types are shown in various load directions corresponding to points in the S-N diagram. Only the most comparable examples from the literature were selected; however, the range of testing parameters is large, making interpretation of the results nearly impossible. One tentative conclusion can be drawn: fatigue life does not depend on load direction, as every fatigue test presented in the diagram was normalised to the static strength.

### 2.5. Size Effect

It is known that the size of the cross-section influences the load-bearing capacity under static bending. Fracture under such loading is quasi-brittle and is therefore modelled according to the Weibull theory of the weakest link. The reasons for the occurrence of size effects in load-bearing timber elements are the natural variability in the wood structure and the presence of knots and resin channels. Logically, the probability of the occurrence of factors that reduce strength is greater with larger volume. This applies to all materials [58,59].

Smith et al. warned that the behaviour of fatigue specimens cannot be transferred to larger elements without considering the size effect [1]. Bonfield et al. did not find a size effect in LVL fatigue tests, but only mentioned a greater probability of this effect occurring under flexural loading [53]. Dimitrov et al. [60] investigated the size effect only to determine the size of the test specimens. They reinforced balsa wood with fibreglass plates and tested specimens of 90 mm and 60 mm width for fatigue. They discovered a size effect in additional tests with 60 mm wide specimens and therefore decided to use wider specimens. However, they argue that it is difficult to determine the size effect using only two sizes. Norlin and Lam [61] similarly tested laminated spruce veneer and found a 3% to 7% increase in strength or resistance due to the size effect. According to the scatter, this increased strength is within error and is statistically insignificant. The size effect in fatigue remains relatively unknown due to the small number of specific studies.

## 3. Influence of Load Type and Shape

### 3.1. Load Ratio R and Mean Stress $\sigma_{m}$

**Figure 20:**
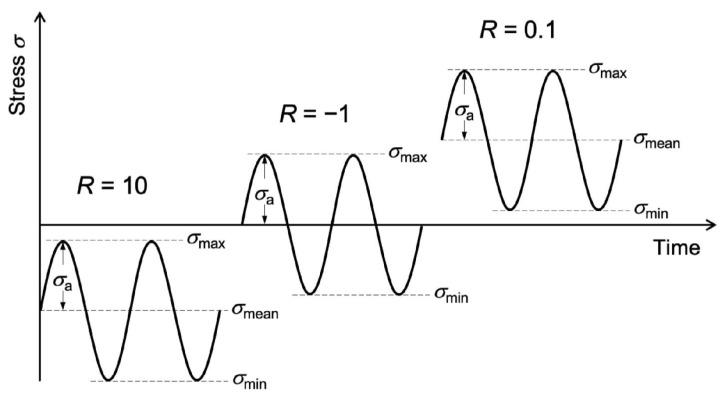
Load ratio and mean stress [45].

The load ratio and mean stress are characteristic and influential parameters in material fatigue. The factor *R* is the ratio between the minimum and maximum stress. The mean stress is the average of the minimum and maximum stress. Tensile fatigue occurs for 0<R<1, while fatigue in pure compression occurs at R>1. Alternating cyclic loading occurs at R<0. The mean stress indicates the stress range, which is negative for pure compression fatigue and positive for pure tensile fatigue. For alternating cyclic loading, the mean stress can be positive, negative, or zero; it is zero for pure alternating loading, R=−1, as shown in Figure 20.

Several different materials respond similarly to changes in mean stress and stress ratio. Pure alternating load is the most destructive for steel and polymer composites, for example [62]. A positive mean stress, i.e., tensile stress, results in a shorter fatigue life for steel. The same dependence applies to wood and wood composites with respect to the stress ratio [4]. By comparing fatigue results in compression and tension from the literature, we cannot conclude whether the fatigue strength of wood-based materials is lower in compression fatigue than in tensile fatigue [12,18,38]. Higher mean stress reduces the fatigue strength of both steel and wood [4,63]. The relationship between the factor *R* and the mean stress is described by the Goodman diagram or constant life diagram. This diagram represents the limit below which the material does not exceed the limit number of load cycles before failure at a given mean stress and stress amplitude ($\sigma_{a}$). For steels, this limit is a straight line, whereas for wood it is more accurately described by the Gerber line, which is a polynomial [1,4]. Using the Goodman line as an approximation is conservative for wood. It has also been found that the failure mechanism changes from a mixed tension-compression mode to a compression mode when the R-ratio is changed from R=−10 to R=10 [37].

### 3.2. Load Cycle Waveform

Long-term loading causes wood to creep and behave viscously, while short-term loading causes it to behave completely elastically. There is no uniform definition among researchers for the boundary between long-term and short-term loading. The waveform of the load cycle plays an important role in fatigue. The three most common waveforms are square, triangular, and sinusoidal. They can be analysed either from the perspective of simulating real loads or in terms of their effects on fatigue. There is no single load cycle waveform that accurately represents the real loading conditions for all structures and products. Furniture is subjected to combined load cycles of triangular and square waveforms, that is, trapezoidal shapes [16]. Bridges are subjected to traffic loads in the form of sinusoidal cyclic loads. Triangular loads are rarely encountered and are not as common in nature as sinusoidal loads. Triangular waveforms are often used for testing purposes, as they provide a good approximation of sinusoidal loads and are easier to handle [53]. Fatigue life depends on the waveform of the loading cycle. Gong and Smith [64] found that square loading is the most critical when the duty cycle is greater than 50%. Bonfield et al. [53] confirm this and also note that the square waveform combines creep and fatigue into a single damage mechanism. Creep and fatigue are two distinct processes of damage formation. Creep also occurs during fatigue, especially at low frequencies, which indicates that wood is a viscoelastic material. Specimens subjected only to creep last longer than those subjected to fatigue, although the accumulated deformation is greater in creep. This suggests that damage accumulates more rapidly in fatigue [64]. The difference between sinusoidal and triangular waveforms is small, but several authors agree that the sinusoidal load shape is more critical [32,64,65,66].

### 3.3. Load Cycle Frequency

**Figure 21:**
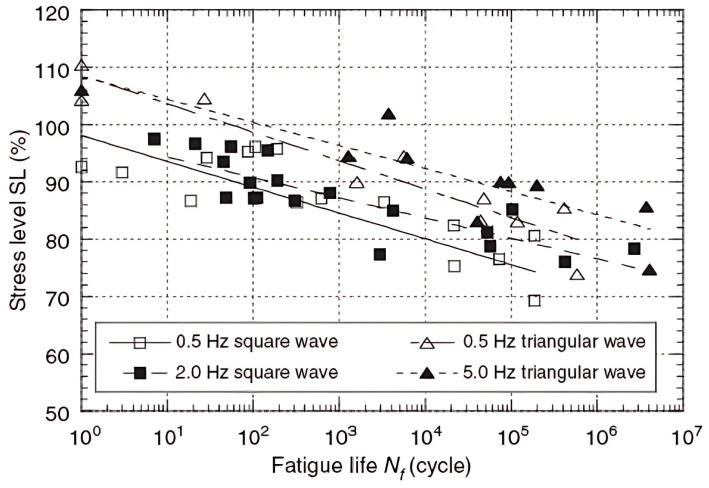
Effect of frequency on fatigue life. Reproduced with permission from [54], Holzforschung; published by De Gruyter, 2014.

Numerous studies have examined the influence of different frequencies on fatigue life across various wood species, including both solid wood and composite materials, as well as in different directions, loading methods, and physical properties [53,54,63,65,67]. The most significant finding is a reduced fatigue life at lower frequencies. Sasaki et al. illustrated this effect in Figure 21, using three different frequencies and two load waveforms. Although the frequency range is small, the difference in fatigue life is substantial.

In the case of low-frequency fatigue, creep predominates over fatigue [68], resulting in a lower fatigue life. When considering the influence of frequency on fatigue at frequencies from 0.1 Hz to 50 Hz or from 50 Hz to 20 kHz, the change in influence is much greater at low frequencies and negligible at higher frequencies [45]. The influence of frequency also changes with the load. Sugimoto et al. [65] found that the difference in energy loss per cycle is much greater at 90% of the static strength than at 50%, where it is practically negligible. This suggests that the influence of frequency is greater at higher loads and negligible at sufficiently low loads, even though the frequency at which this was found, 5 Hz, is relatively low. The influence of frequency also depends on the moisture content. If the moisture content of the wood is high, the influence of changing frequency on fatigue is significant, whereas it is negligible if the moisture content is low [39]. Sekhar and Shukla conducted experiments at a frequency of 23.7 Hz [1], at which the temperature increases due to internal friction and the moisture content decreases [23], resulting in an apparently better fatigue life. Salmen found that at lower frequencies, i.e., 0.5 Hz, the influence of high temperature on fatigue damage is much greater than at higher frequencies [32]. Fatigue frequency is therefore a very important factor when comparing the results for the same type of wood, which is why almost all authors on this topic specify it as a test parameter.

## 4. Wood and Wood-Based Materials

### 4.1. Solid Wood

We have already established in Section 2.4 that wood is a heterogeneous and anisotropic material. Its response to fatigue is difficult to standardise even within a single wood species. Therefore, pure solid wood is used for the tests, i.e., without knots, cracks, or resin channels. Samples are taken from the same part of the trunk to reduce variations in density. When preparing the samples, attention must be paid to the orientation of the fibres, as this has a significant influence on the fatigue response. Despite eliminating factors that influence variability, the scatter remains large.

In practice, especially with solid wood, it is rare to find conditions where such high-quality pieces of wood can be used as in the tests. Therefore, Klemenc and Fajdiga developed a statistical model based on the two-parameter Weibull probability density distribution to predict fatigue life as a function of load, taking into account density, sample series, and orientation. They tested the fatigue life of specimens loaded in bending in both radial and tangential directions [15]. Their model successfully accounted for variation in density and orientation (radial/tangential), demonstrating the potential of the method to include additional influential factors.

Fatigue life is largely independent of wood species [45]. A comparison of the results from Schonbauer et al. and Bonfield et al. confirms that fatigue life does not depend on wood species [37,45]. Kandler et al. reconstructed knot geometry using laser scanning based on geometric features visible on the surface [69]. The reconstructed knots are used in analyses with the finite element method (FEM) to predict reduced strength [58,70]. Kretschmann investigated the influence of knots on fatigue and found that fatigue life is lower in the presence of knots [38]. Knots in wood represent stress concentrations, specifically tensile stresses perpendicular to the grain. The fibres in knots are oriented differently from the global fibre direction, which reduces strength. The density is higher in knots, increasing fracture energy [71]. Specific studies on the influence of knots on fatigue are scarce, so their effects can only be speculated upon. Considering that knots represent a deviation of fibres from the global alignment, and based on static test results, it can be assumed that fatigue life is lower in the presence of knots. However, there is also an explanation and possibility that this may not be the case, as the higher density of knots could mean a higher overall density and consequently a longer fatigue life. When testing Japanese cypress with drilled holes, the fatigue life was even higher than in specimens without holes [4].

### 4.2. Engineered Wood

Engineered wood is divided into two main categories based on intended use: beams and panels or walls. The most common forms of engineered wood used for beams are LVL, Parallel Strand Lumber (PSL), Laminated Strand Lumber (LSL), and Glue Laminated Timber (GLT). For panels, the most common forms are cross-laminated timber (CLT), oriented strand board (OSB), particleboard (PB), medium density fibreboard (MDF), and plywood. These forms are preferred because they offer better dimensional stability, weather resistance, and higher strength or stiffness, which is consistent in several directions [72]. A comprehensive review of the fatigue response of these and similar forms of engineered wood would be extensive and would require a separate study, which was not conducted in this work.

It is not possible to produce wide or long structural elements from a single piece of solid wood, nor to create complex shapes. By using engineered wood such as GLT, LVL, or OSB, any shape and size can be achieved. Their mechanical properties are more homogeneous because individual pieces of wood with similar properties can be combined. This is reflected in the higher static strength but may not be reflected in longer fatigue life.

Researchers do not yet agree on whether the fatigue life of solid wood and engineered wood is approximately the same. Tsai and Ansell, based on a literature review and their own tests, found little difference in fatigue life between LVL and solid wood [4]. The results of Schonbauer et al. and Bonfield et al. [45,53] indicate the independence of the fatigue life of solid wood and engineered wood. On the other hand, some studies have found the opposite [73,74,75]. This discrepancy is due to the various manufacturing processes and adhesives used for engineered wood [76]. There is no consensus on the effect of adhesives–whether they improve fatigue life or have no effect [4]. The adhesive between the elements is usually stronger than the base material. The adhesion and cohesion of adhesives are stronger than the matrix in the wood, i.e., the lignin. With good adhesives and high-quality wood elements, a stronger structural element can be produced that is more resistant to fatigue. Gaff and Gašparik [76] investigated the modulus of elasticity of LVL and solid spruce wood and found that the modulus of elasticity of both decreases with an increasing number of load cycles, with this effect being more pronounced in solid wood. This indicates greater damage in solid wood due to fatigue. In general, engineered wood had a higher modulus of elasticity.

### 4.3. Composite Materials

Wood-based composites are classified into two groups: homogeneous and inhomogeneous. In homogeneous composites, wood is present as small particles or even nanofibres, together with another component of the composite material. Examples of homogeneous wood composites include wood polymers [77] and wood cements [72].

Inhomogeneous composite materials combine the positive properties of two or more materials in their primary form of use. Examples include composites made of timber and concrete [78], wood reinforced with glass fibres [60], and composites made of wood and aluminium [49].

A comprehensive review of their fatigue properties would require a separate study. Nevertheless, we mention some examples of composite materials and their response to fatigue. Yeoh et al. investigated the behaviour of a composite of timber and concrete [79]. After 2 million load cycles, no obvious loss of stiffness was observed in the rectangular notch-connected floor beams. Davids et al. examined the response of used beams reinforced with glass fibres [80]. Similarly, after 2 million cycles, they did not observe a significant reduction in the static strength of the Glulam timber beams. Yang et al. studied the response of a wood–polymer composite to fatigue [81]. As expected, wood–polymer composites exhibit greater predictability than solid wood.

## 5. Measurement Methods and Models for Predicting Fatigue Response

For timber constructions, we aim to optimise raw material consumption while ensuring safety. Currently, the design of structures according to the EN 1995-2 [8] standard considers the fatigue of elements subjected to bending, tension, or shear, as well as connections with dowels or nails, based on a criterion that depends on the maximum design stress amplitude, material strength, and the safety factor. The calculation according to the standard assumes a cyclic load with constant amplitude. This approximation is unfavourable for structural optimisation. Based on mechanical loads and relevant influencing factors, we seek to predict fatigue life in addition to static load-bearing capacity. To this end, many models have been developed to describe the stress-strain state under cyclic loading and to predict fatigue life. Generally, these are divided into analytical and experimental models. Analytical models use mathematical descriptions to represent the physical state, with results typically verified by experiments. Experimental models are based on experimental data and describe the physical state using mathematical expressions that incorporate measured variables. Experimental data are obtained using various measurement methods, techniques, and devices.

### 5.1. Measurement Methods

During the fatigue process, we physically monitor force and displacement. In constant force mode, the force must remain constant, regardless of the magnitude of displacement. This alters the stress, which is determined from the measured deformation and the modulus of elasticity or shear modulus. In addition to the basic measurements, we also identify other measurable influencing factors in wood fatigue, such as detection and measurement of crack length, monitoring of natural frequency, and dissipated energy, which enable us to predict when the product is approaching the point of failure.

- Acoustic emission
  As a non-destructive measurement method, acoustic emission (AE) is the most common technique for crack detection. It can detect low-energy events and, therefore, very small cracks. An AE event is defined as an acoustic signal whose amplitude exceeds a threshold value. AE is a useful method for detecting cracks in various materials such as concrete, steel, wood, and composites [82], and is used in various industries [83] and in construction [84]. In wood and wooden structures, AE is used in several cases, including during the mechanical processing of wood, during drying, in the event of damage, and to monitor the mechanical condition of structures [85].
  The use of AE in fatigue monitoring is not widespread. Chen et al. [44] compared the number of events recorded by AE and their temporal evolution under static torsional loading of spruce and beech wood, as well as the number of events under cyclic loading for both species. They found that more events occurred during fatigue than during static loading, indicating a greater number of cracking events under fatigue. The cumulative number of events was higher during the fatigue of spruce wood, and the rate of event occurrence was also greater, resulting in a lower fatigue life compared to hardwood.
  This method is useful for monitoring crack formation during fatigue and has the potential to measure crack length by determining the position of the crack tip. The crack length can be calculated from the wave propagation time from the starting point to the detector. Based on the critical length, the lifetime of the material can be predicted.
- Ultrasound
  Ultrasonic waves can be used to stress a structure [86], or to measure the dynamic modulus of elasticity [10,11]. There is a difference between the static and dynamic modulus of elasticity. Due to viscoelasticity, the dynamic modulus of elasticity is higher. This is because the duration of the loading cycle is very short when using ultrasonic waves—1 µs (1 MHz) [10]—whereas with static loading, the duration is longer—1.5 min [48]—which results in a stronger viscoelastic influence. Measuring the dynamic modulus of elasticity with ultrasonic waves has disadvantages. The method is not suitable for longer samples because the wave has a small amplitude and a high frequency. The most important data used to calculate the dynamic modulus of elasticity are the wave propagation times, which represent an average wave propagation speed. In the case of cracks, knots, or resin channels in the sample, the disturbance bypasses them and does not reflect the true modulus of elasticity. This method has not yet been used to monitor the fatigue response of wood materials.
- Digital image correlation
  Digital image correlation (DIC) is a non-contact, non-destructive method for measuring deformations. This method is often used in the field of wood [46,70,87,88] and other building materials [89,90]. Displacements or deformations are determined by comparing two consecutive images. Individual pixels in the images are compared, and correlation algorithms are used to determine the displacements between the two images. The samples must have sufficient surface texture, or it must be created, for the measurement to be successful. The images can be acquired with a standard or high-speed camera and processed with a correlation algorithm after acquisition. Real-time image processing at 30 or 60 Hz would require significant computing power and would be impossible with a high-speed camera. In the case of wood fatigue, the frequencies are usually 10 to 15 Hz, which means that data must be captured at a frequency of at least 20 or 30 Hz according to the Nyquist rule. With current technology, it is not possible to monitor deformations during fatigue at such a frequency in real time; they can only be analysed afterwards. The measurement method is suitable for monitoring structures where it records deformations due to stochastic loads. There are few research papers that monitor deformations during the fatigue process using the DIC technique. One such study is by Chen et al. [91], who used DIC to study the fatigue of an OSB panel with cyclic bending loading at different relative humidity values. Using DIC, they were able to accurately measure the swelling of OSB due to higher relative humidity and its effect on deflection during fatigue loading.
- Linear variable differential transformer
  TThe linear variable differential transformer (LVDT) converts linear movement into an electrical signal. It is most commonly used to measure the displacement of various materials under load. It is also employed in static bending, tension, and compression tests on wood. Its application is not limited to displacement measurement; it can also measure pressure, force, current, and other physical quantities. The LVDT is valuable when high resolution and accuracy, ease of installation, and real-time data acquisition are required. However, its limitations include high cost and a restricted measurement range [92]. It is a reliable, simple, and proven measurement method, which is why it is also used in fatigue tests [52,93].
- Strain gauges
  Strain gauges measure deformation and are commonly used in the field of wood and wood composites [44,94,95,96]. The heterogeneity of wood leads to greater variability in measurements with strain gauges, as these only cover a small area. In comparison, DIC covers a larger area and partially reduces the influence of heterogeneity, resulting in less scatter in measurements [97]. Strain gauges are used for both static and fatigue tests without any major shortcomings or weaknesses.

### 5.2. Models for Predicting Fatigue Response

Clorius et al. [12] investigated various models for predicting the fatigue life of wood. The main approaches for quantitatively describing wood fatigue are based on either the number of cycles to failure or the duration of loading to failure. In the first approach, the number of cycles to failure is recorded at different stress levels, resulting in a Wöhler curve, which is well known for steels. In the second approach, the accumulated time under load at different stress levels is recorded, producing a Madison curve. For wood, neither a complete description using the number of cycles nor one using the accumulated time under all conditions is accurate. Therefore, alternative models are used to represent the fatigue response based on either the number of cycles or the time to failure.

- Damage accumulation models [39,98,99]
  These use the damage variable α, which takes values between 0 and 1. This variable is a function of time, so despite differences in notation, these models only represent time-dependent damage. They do not account for load oscillation, which is characteristic of fatigue.
- Energy models
  These take into account the time-dependent properties of wood. The energy consists of time-dependent stress and strain. Strain can be divided into elastic, viscous, and viscoelastic components, so the energy model can be formulated with any combination of these properties. Damage is determined by the critical value of the accumulated energy from the loading cycles. The authors of these models encountered problems such as the additional work caused by reverse deformation during creep and the release of stress at failure. The energy generated by elastic deformation at failure should not be considered, as it is return energy that does not contribute to damage.
- Number of cycles
  The fatigue strength is determined by counting the number of load cycles. Linear fracture mechanics are assumed, which also implies linear crack growth. For a constant amplitude of load cycles, Paris’ law is applied; it predicts linear crack growth and relates the crack growth rate to the stress concentration factor. The growth rate is defined as the change in crack size per cycle. The number of cycles to failure is determined based on the critical crack size. Paris’ law is successfully used in complex fatigue cases, such as modelling the formation and propagation of a fatigue crack on the surface of a gear tooth [100]. For variable stress amplitudes, the Palmgren-Miner rule is used, which sums individual load cycles as a percentage of the fatigue life at the corresponding stress levels. The cycle count methods do not consider the duration of individual cycles and therefore do not distinguish between the effects of low and high frequencies.
- Damaged viscoelastic material (DVM)
  This assumes an initial crack of length *l* in the material. The damage variable is the energy released during crack propagation. The model was originally developed for static loading and has been extended to include fatigue [101,102]. It considers both elastic and viscoelastic deformations, taking into account frequency and capturing the number of cycles [12]. Among fatigue life prediction models, the DVM best adapts to the viscoelastic effects in wood fatigue [101].

Fatigue response prediction models are summarised in Table 1. Damage accumulation models and number of cycles models are simple approaches that rely on linear damage accumulation and crack growth. Energy models and the DVM account for the viscoelastic effects of wood and are more accurate in predicting the fatigue response. The DVM combines time-dependent and oscillation effects from cyclic loading, making it the most applicable. However, it is valid only if an initial crack is present.

In addition to the models described, other models for describing fatigue are based on experiments, analytical approaches and/or numerical methods [12,15,60,103]. Some of the listed models require numerical calculations. Numerical models are always used to simulate fatigue response.

### 5.3. Stress–Strain Simulations with Cyclic Load

Simulations enable the analysis of how structures with complex geometry respond to various loading conditions. They can be used to predict a product’s fatigue life quickly and easily. Based on simulation results, decisions are made regarding changes in design or loading. In this way, products are optimised to be lighter and safer. Fatigue is often the cause of structural failure. Research is therefore being conducted into the best methods for simulating such load conditions, including for wood materials.

Damage is the most commonly used criterion in simulations to determine the point of failure. It is described by mechanistic and phenomenological models. Mechanistic models quantitatively describe the progression of damage and are suitable for a variety of different materials and loading methods. Phenomenological models describe the remaining resistance and fatigue life based on macroscopic observations. Many phenomenological models require a large amount of experimental data [104].

There are already various damage criteria and failure models for polymers and polymer composites with different reinforcing fibres, such as the Tsai-Wu failure model, which is a general theory for anisotropic materials [105], and the Hashin damage criterion [106]. Tsai-Wu and Tsai-Hill are criteria developed for anisotropic materials, similar to von Mises for isotropic materials [107], meaning they are generally applicable to wood, at least under quasi-static loading. The weakness of these two criteria is that they do not consider the reduction in strength after the first damage occurs. However, the Hashin-Rotem damage criterion accounts for four types of damage: fibre fracture in tension, fibre buckling in compression, matrix failure in tension, and matrix crushing in compression [107]. It was developed to describe the fatigue of polymer composites with reinforcing fibres. For this reason, it would also be suitable for describing the fatigue of wood, as the structure of wood is similar to that of fibre-reinforced polymer composites.

In our literature review [108,109,110,111,112,113], there are several examples of three-dimensional constitutive models for simulating wood fatigue, as well as models for simulating composite materials. One such three-dimensional constitutive material model for wood, which can describe the stress-strain state under variable-amplitude cyclic loading and account for different failure modes, uses a coupled general orthotropic plastic model with isotropic hardening and an isotropic continuum damage model. The model separately considers the orthotropic elastic response, isotropic damage, and orthotropic plastic response. The elastic response is described by Hooke’s law using a fourth-order orthotropic compliance tensor. Damage is treated as isotropic to preserve the symmetry of the Cauchy stress tensor. Non-linear damage evolution is modelled within the framework of continuum damage mechanics and the theory of irreversible processes. In particular, the model defines damage criteria and criteria for damage evolution in tension and compression. Plasticity is included by incorporating isotropic hardening. The entire model was verified by uniaxial tension and compression tests, as well as by cyclic load tests on steel dowel connections, the response of which was also simulated using the finite element method (FEM) [108].

The experimental data required by the model are the elastic and shear moduli, as well as the Poisson’s ratios for all three directions. To model the damage, the model requires the value of the energy released between the point of maximum stress and the point of complete failure. This energy value is proportional to the energy density at failure, which varies depending on the loading direction and the type of failure. The calibration factors are determined using monotonic and cyclic tests under compressive load.

Another example [109] of a three-dimensional model for the non-linear analysis of wood was developed for a half-lap joint subjected to cyclic loading with increasing amplitude. The model considers damage and damage evolution separately in tension and compression. Like the previously mentioned model, it uses the Hill criterion for damage, while for damage evolution it employs a different, more recent model [114]. Damage evolution is modelled with exponential strength degradation. In contrast to the previous model, this model specifically accounts for the influence of wood viscosity by using the viscous regularisation technique to ensure stable convergence of the numerical model, which includes a time component in the fatigue description. The parameter values in the equations are determined from the mechanical properties of the material. The model requires all elastic and shear moduli, as well as the energy released during tensile failure.

The necessary steps for a successful and accurate fatigue simulation include selecting the damage criterion and the rheological model. The experimentally determined σ−ε curve and the mechanical properties of the material must be known. For damage accumulation, a modified Paris law or the Palmgren-Miner rule for linear damage accumulation is used. A simpler method for determining the damage is the Rayleigh approximation, which is only applicable under a Gaussian distribution of the amplitudes of the load cycles [115].

Reduced strength or resistance are important criteria in simulation, as they determine the remaining static strength and predict fatigue life. Brautman was the first to attempt to describe linear strength reduction [115], and since then, several authors have investigated equations for nonlinear strength reduction. Some commonly used models for strength reduction in wood fatigue simulations are cohesive zone models. These account for the nonlinear growth of damage in quasi-brittle materials such as wood and concrete [103]. They combine damage mechanics and linear-elastic damage mechanics, and are successfully used in numerical simulations with finite elements of zero wall thickness [116]. Dourado et al. compared the results of numerical simulations and experiments in mode I damage of maritime pine (Pinus pinaster). They found that a simple bilinear cohesive zone model using a modified Paris law consistently described the fatigue damage in this loading mode. The simulation results also agreed with those from the experiments [103].

Any fatigue life prediction model requires experimental data. In addition to the equations and input data, an automated system is needed to calculate these equations for individual finite elements and to visualise the results. For more complex geometries, commercial software packages such as ABAQUS (SIMULIA Abaqus) - 2025 or ANSYS 2025 R2 are usually used, while for simpler two-dimensional geometries, Wolfram Mathematica version 14.3 or Matlab R2025a are employed. Recently, major simulation software providers have also begun to offer fatigue modules for composite materials, including wood composites. These providers include Altair with the stand-alone ESAcomp (Altair) - 2025 module [117], and Magna FEMFAT 2025 with the FEMFAT laminate module for the simulation of fatigue of fiber-reinforced polymers (FRP) [118].

## 6. Areas and Main Causes of Fatigue Failure

Recently, the trend of using wood and wood composites has become fashionable again. Wood offers a favourable ratio of specific strength to carbon emissions. Its high specific strength makes it suitable for constructing lightweight structures. Wood is also increasingly valued for its aesthetic qualities. Studies have shown that the natural appearance of wood in office spaces reduces stress levels [119]. Due to the current increase in the use of wood, greater attention must be paid to wood fatigue, as our understanding is insufficient to optimise structures for minimal raw material consumption while maintaining adequate safety. Historically, wood has demonstrated good fatigue resistance, but its behaviour in this regard is highly unpredictable. Below, we provide an overview of the main areas where wood has been, is, and could be used.

### 6.1. Buildings

In recent years, structural wood has been used more frequently in construction because it has a lower carbon footprint than concrete and steel. This has raised new questions about its response to vibrations, as timber structures are lighter and less rigid. The main causes of cyclic loading in buildings are wind and earthquakes.

- Walls
  The walls are the most heavily loaded elements during wind or earthquake events. Timber buildings are light and flexible, which poses a problem in terms of fatigue. A gust of wind or a seismic wave causes the building to sway, and it does not immediately return to its original position but oscillates for some time. During this period, the walls are repeatedly subjected to shear stress. For this reason, dampers are installed in buildings, particularly in multi-storey structures, to reduce vibrations caused by such events. These dampers are often made of steel, but environmentally friendly alternatives are being researched. Wu et al. [120] tested the durability of variable vibration dampers made of wood in a seismic simulation. They found that these dampers successfully reduce vibrations and provide satisfactory energy dissipation. In the event of failure, they can be replaced with new ones.
- Roofs
  The main loads acting on roofs are distributed loads such as snow and wind. Of these two, only the wind load is cyclical over a short period. Timber is used in roofs as trusses and panels and is therefore not subject to significant cyclic loading. As with other building components, the most critical load on the roof is at the connection between the wall and the roof, and between the roof and the ridge beam. Alhawamdeh and Shao [121] tested three different connections between the wall and the roof: RTWC (Roof-To-Wall-Connection) with nails, and two different elastomeric adhesives. They found that adding adhesive to the nail joint increased durability by 250% to 330%. The nail-only connection is suitable for structures in areas with low wind speed amplitudes.
- Floors
  Aloisio et al. [122] focused on floors and buildings from the perspective of vibrations. Where vibrations occur, signs of fatigue can also be expected. Wooden floors are found in residential, commercial, sports, and public spaces. Static loading by one person or even a group does not affect the strength of the floor. However, the load amplitude can increase when a group is moving (such as during performances, events, or dances), which may lead to fatigue and a subsequent reduction in strength. According to EN 1995-2 [8], the natural frequencies of the floor must be checked during construction. Human walking has a frequency of 1.5 Hz to 2.5 Hz. It is recommended that floors have a natural frequency at least four times higher to avoid possible effects of higher harmonics. If the floor resonates with the load, the deflection amplitude could reach destructive levels. Conversely, even if the floor is not in resonance, significant fatigue loading may still occur.
- Windows
  Windows represent a stress concentration in the wall that must be considered when designing building walls. Rather than being a weak point, a window can be designed as a load-bearing part of the wall [123]. In his research, Seitz [124] compared the response of walls with and without openings to cyclic loading according to the CUREE protocol. He found that wall stiffness decreases with the presence of an opening. He also compared walls with openings reinforced with steel straps at the edges. He found that such walls exhibit a smaller decrease in stiffness and higher strength. The reduction in strength is more pronounced than in a wall without an opening. Generally, energy dissipation is greater for a wall with an opening and reinforcements. Fajdiga et al. [125] developed a test rig for fatigue testing of long, slender window frame elements and tested it on a single window frame sample. The frame was made of a composite material consisting of spruce wood and an aluminium alloy reinforcement. They found that spruce wood is less sensitive to fatigue than typical metals.
- Joints
  Joints are widely used in the construction industry. There are various types of joints, ranging from simple joints with nails or screws to complex joints that provide vibration damping. Joints use steel connectors, which may consist of nails and screws or steel plates and angles fastened with nails and screws. Fatigue affects both wood and steel connections. For roofs, we have already described the results of the study by Alhawamdeh and Shao, who tested the joint between the roof and wall. Steel plate connections are commonly used in building construction. Ling et al. [88] investigated their use, properties, advantages, and disadvantages. They found that under simulated seismic loading, the strength of the connection did not decrease, but its stiffness decreased significantly. After simulated cyclic wind loading, increased stiffness of the joints was observed, while the strength did not decrease significantly. In his study, Richardson [126] described the tests and results of static and dynamic mechanical loading of three different joints: a joint with a steel plate, a joint with a cover plate, and a half-lap joint. Under dynamic loading, he found that higher loads were possible with the steel plate connection. The connection with a cover plate had lower shear stiffness after dynamic loading, while the other two connections had higher shear stiffness. Madhoushi and Ansell [127] compared the fatigue life of LVL connected to GFRP-bonded bars. They found that LVL had higher static strength and durability, but lower energy dissipation under cyclic mechanical loading. Joints are not only used in construction; a large proportion are also found in furniture, which is exposed to even more frequent cyclic loads.

### 6.2. Furniture

The effect of cyclic loading on the durability of furniture has been studied since the earliest times. Ratnasingam et al. [16] state that furniture was initially dimensioned for load-bearing capacity. Manufacturers aimed to produce more robust and durable pieces, prioritising these qualities over aesthetics. Seating furniture (chairs, armchairs, sofas, beds, etc.) is subject to the greatest cyclic loads among all types of furniture. These items experience cyclic stress from normal loads, such as a person’s weight, and are occasionally subjected to increased loads (rocking on a chair, jumping on an armchair or bed, or several people using a single piece of seating furniture). Over time, such overloading typically leads to failure, as the strength decreases to a point where the furniture can no longer withstand the increased load [16]. According to one design guideline for furniture, components are dimensioned to one third of the static strength and joints to one quarter of the static strength.

Many furniture parts are exposed to cyclic loads throughout their lifetime. Those used daily have a higher probability of fatigue failure. Therefore, it is not sensible to design them for only a fraction of the static strength. It is preferable to design for fatigue, which requires experimental data to model fatigue behaviour. Fatigue of wood is influenced by many factors, so a model based solely on tests of wood or joints is insufficient. Data on the fatigue of the entire piece of furniture under the intended load are needed. The first proposed models used incremental cyclic loading. The problem with these early models and tests was that they applied cyclic loading under controlled displacement, which does not reflect real conditions, as stress decreases due to creep. Later, numerical FEM models were introduced. Yildirim et al. [128] compared experimental and numerical results when testing and analysing a simple chair. They used experimental data from a previously determined S-N curve for red pine in the simulation. They found that the numerical model accurately predicted the critical failure points and safety factors. This approach represents the future of furniture design, as it avoids lengthy and expensive testing procedures. In addition to typical pieces of furniture such as chairs, tables, and worktops, hinges and handles in cupboards and drawers are also subject to fatigue.

### 6.3. Bridges

The construction of wooden bridges is increasing. By 2013, 817 wooden bridges had been built in Sweden, of which 501 were for pedestrians and 316 for traffic. This represents 25% of all bridges built during this period. Wooden bridges have three main disadvantages: lower durability, fire risk, and frequent maintenance [122]. The recent collapse of the Tretten Bridge in Norway, where the cause has not yet been confirmed, demonstrates that predicting the behaviour of wood remains very difficult. After reviewing the facts and evidence, it is believed that one possible cause of the collapse was a decrease in strength due to fatigue [129]. The introduction of Structural Health Monitoring (SHM) could help prevent such events. The use of SHM in timber bridges remains low, mainly because there are no developed systems to enable this.

Davids et al. [80] simulated the fatigue of wooden bridge beams reinforced with glass fibres on the tension side. They simulated 50 years of service using accelerated ageing, involving cyclic changes in moisture content and cyclic mechanical loading. They observed how annual variations in moisture content affect the bond between the glass fibres and the wood, and how these changes influence fatigue. The tests were conducted on 6.7 metre long beams to replicate realistic bridge conditions. Cyclic loading was applied under displacement control. After two million cycles, none of the test specimens failed. Following fatigue testing, quasi-static tests were performed, during which all three failure modes were detected: most specimens failed in tension, fewer in compression, and the fewest in shear. It was found that after two million cycles, no significant decrease in strength occurred in specimens subjected to 50 years of simulated moisture cycling or in those with intentional delamination in the fibreglass layer.

In recent times, laminates have been used primarily in bridge construction, as they offer greater strength and resistance to environmental influences. Bridges are categorised as either beam bridges or panel bridges. Tazarv et al. [130] confirmed that wooden bridges are a cheaper alternative to concrete bridges and do not lose strength under fatigue. They tested up to half a million cycles, which corresponds to a service life of 50 to 90 years if the bridge is built according to the requirements of the AASHTO (American Association of State Highway and Transportation Officials). The cases studied by Sasaki et al. demonstrate the seriousness of bridge fatigue. They examined 12 concrete bridges with five T-beams each. After about 40 years, damage occurred in both the concrete matrix and the steel reinforcement [131]. Cracks appeared in the concrete, and the steel matrix showed signs of brittle fracture, which is typical of fatigue. With this comparison in mind, it can be confirmed that wood has good resistance to fatigue loading.

### 6.4. Power Transmission Poles

Power transmission poles with larger spans are steel skeleton structures, while those with smaller spans remain ordinary wooden poles or A-poles. They are subjected to cyclic loads from gusts of wind, which act directly on the poles and indirectly through the cables. As lightweight and slender structures, they exhibit greater flexibility and low damping. During earthquakes and wind gusts, they vibrate at their natural frequency [132]. Wooden poles offer several advantages over steel or concrete poles. Their lighter weight makes them easier to transport and install, and their flexibility increases their resistance to earthquakes and wind gusts. They are used for around 45 years, whereas concrete poles, for example, need to be replaced every 25–35 years. Their main weaknesses are lower strength and poorer weather resistance [133]. To date, research on wooden poles for power transmission has focused on predicting reliability after a certain period of operation and determining when replacement is required based on strength assessment [134,135]. Specific research examining the impact of fatigue on these structures, or at least verifying it, has not yet been conducted. It would be useful to investigate whether the strength of the poles is reduced more by cyclic loading or by natural decay and decomposition due to the low resistance of wood to moisture.

### 6.5. Marine

Long ago, when wooden ships sailed the oceans, fatigue was already known, but it was defined as the time under load, so ships were designed to about 50% of their static strength [1]. Until the 1970s, wood was still used for shipbuilding in some regions, but since then ships have been constructed from composite materials, as these can achieve lower weight with the same strength, resulting in reduced fuel consumption and higher speeds [136]. To date, several ships have been damaged or sunk simply due to repeated wave impacts and other stresses during voyages [137,138,139]. Today, monitoring systems are used on ships carrying bulk cargo to detect fatigue damage before it reaches a critical level [140]. In shipping today, wood is used only for small boats and piers where wave loads are too low to cause fatigue damage. In boatbuilding, wood is sometimes used as part of a composite material. In this case, it is more resistant to fatigue than the polymer component of the composite material [141].

### 6.6. Vehicles

There is increasing pressure in the automotive industry to use renewable and recyclable materials. Due to its favourable mechanical properties, wood is a highly attractive, environmentally friendly alternative to polymers. The first significant attempt to use wood as a structural component in a car was realised in the working concept of the NIOS car [142]. In contrast, the Morgan Motor Company has used ash wood in its vehicles for 100 years [86]. The use of wood was more common in smaller aircraft. During the Second World War, one of the fastest aeroplanes was the De Havilland Mosquito, which was largely constructed from various types of wood [143]. The fact that most damage in aeroplanes is due to fatigue, up to 55% [144], indicates that wood, in addition to its favourable mechanical properties, is also highly resistant to fatigue.

## 7. Conclusions

We reviewed the current work, research, findings, and results in the field of wood fatigue. Density and moisture content are the most important influencing factors in static strength testing, and they play an equally important role in fatigue. Other factors influencing fatigue include fibre orientation, mean stress, R-ratio, load cycle waveform, frequency, and temperature. Many of these influencing factors are interdependent, and their effects are shown in Table 2 using a colour scale. The factors are divided into physical properties and loads. Some properties and loadings have two states, for example, low density (LD) and high density (HD), or low mean stress (LMS) and high mean stress (HMS). Combinations of different states of physical properties and stresses are indicated by abbreviations, such as the combination of low density and low mean stress (LD-LMS), where the influence of the combination is indicated by a colour, as shown in the table legend. The evaluation criterion ranges from very unfavourable to very favourable. The descriptive states correspond to quantitative values from 0 to 1, from which we have obtained an evaluation of the influence of the individual factors. The quantitative evaluation of the influence is determined from the difference between the average values of the individual states of the physical properties or loads. For example, the overall rating of the influence of density is the difference between the average value of the rating at low density and at high density. Similarly, the overall influence rating is also determined for mean stress, which has the highest value, confirming the appropriateness of the influence rating method.

Throughout the review, we discussed research by many authors who used various wood species, engineered wood, and composites in fatigue tests to determine fatigue strength, investigate influential factors, and develop prediction models. We examined the equipment and methods used for collecting experimental data, as well as the models used to predict and simulate fatigue life. Finally, we identified areas where fatigue is the main cause of failure and noted that wood has good resistance to fatigue.

### 7.1. Findings

The most important findings on the response of wood to cyclic loads under various influencing factors are shown in Table 2. Additional findings that determine approaches for addressing the response of wood to cyclic loads are as follows:

- The σ−N curve is monotonically decreasing up to $10^{9}$ cycles [45].
- The researchers set the fatigue strength at $10^{7}$ cycles, for softwood (spruce, fir and Japanese cypress) it is 15–35% of the static strength [4].
- The energy loss per load cycle is initially higher, then decreases in a few cycles and remains approximately constant until shortly before failure, where it increases exponentially in just a few cycles [66].
- Due to the influence of frequency, the waveform of the load cycle and the time under load must not be neglected.
- The square waveform of cyclic loading is the most destructive [64].
- The Goodman line describing the relationship between $\sigma_{m}$ and $\sigma_{a}$, is conservative for wood, the relationship is better described by a polynomial Gerber line [1,4,7].
- At sufficiently low load frequencies (<0.5 Hz), the influence of creep begins to dominate over fatigue [67].
- Damage from creep occurs earlier than damage from fatigue if the criterion is time under load, while the accumulation of damage from fatigue occurs faster [67].
- Knowledge about fatigue cannot be transferred directly from tested samples to structural elements due to the size effect [1].
- The deviation of the load angle from the fibre direction should not exceed 10° for elements subjected to fatigue [1].
- The process of quality control and the classification of wood into strength classes can damage the wood and reduce its resistance to fatigue [1].
- The dependence of fatigue on the frequency of cyclic loading is greatest in compression and least in bending.
- At lower frequencies, up to 100 Hz, the influence of frequency on fatigue is greater, while at higher frequencies, such as 1 kHz, it is negligible.
- It is not yet clear if engineered wood is more resistant to fatigue because it has the positive properties of solid wood and greater homogeneity.

The findings presented in Table 3 are a direct result of the literature review and additionally summarise the effects of frequency, temperature, and load direction in relation to moisture content and heat generation on fatigue strength.

### 7.2. Research Opportunities

After reviewing the literature on wood fatigue, we have identified areas that remain under-researched. Temperature does not have a significant direct effect on fatigue strength, but it can have a significant indirect effect on other properties. Currently, there is little research that isolates changes in moisture content during fatigue testing at different temperatures, so the direct effect of temperature remains unknown.

There are many combinations of load directions, forms, and types of cyclic loads. We have found that pulsating cyclic tensile loading in the direction of the grain is poorly researched. The damage mechanism in the direction perpendicular to the grain under cyclic tensile loading is insufficiently described. The damage mechanism in this direction under compressive loading is also insufficiently described. The main disadvantage of all combinations is that it is impossible to compare results between different types of loading, as different test conditions are used. There is a lack of a basic comparison of the direction, shape, and type of cyclic loading for each type of wood under the same test conditions; in other words, a standard for testing wood for fatigue is needed.

In terms of practical applicability, the effect of structural element size on fatigue has not been sufficiently studied. There is a lack of robust models that accurately transfer fatigue results from specimens to structural elements, considering the influence of size and, consequently, inhomogeneity. Some studies have examined the effect of knots on fatigue, but the findings are inconsistent. It remains unclear how higher density near knots and fibre angle deviation affect fatigue. The impact of missing knots is also unknown. The presence of a hole may represent a stress concentration that either improves or worsens response on fatigue.

When examining specific examples of wood used for structural purposes, it becomes apparent that most structures and products are not designed with fatigue life in mind. Structures for which fatigue life has not been modelled should be monitored through the implementation of SHM systems. There are few devices and models available for implementing SHM in this field. One possible application of such a system, besides bridges, is for poles used in electrical energy transmission. For these poles, it is not known whether deterioration results from environmental degradation or a reduction in strength due to fatigue.

### 7.3. Future Work

Certainly, unexplored areas themselves suggest directions for future work. Although our overview is comprehensive, it does not cover all aspects. A separate study on the fatigue of different types of glulam and wood composites compared to solid wood is needed. Further investigation into the fatigue of joints would also be valuable. There are many potential applications in the field of measurement methods. In particular, it would be beneficial to test the integration of sensors. For example, ultrasound could be used to measure the dynamic modulus of elasticity under cyclic loading, while deformation or stress could be measured using the DIC method. Most importantly, wood fatigue testing methodology should be standardised, including predetermined specimen size ratios, waveforms, frequencies, load ratios, load directions (including specimen fixtures), moisture content, and density classes.

This review of the fatigue of wood and wood-based materials aims to provide future researchers new to the field with a comprehensive overview of influential factors, materials, prediction methods, and areas where wood fatigue occurs, including the main causes. From this, new research may emerge, and expanding research on wood fatigue will contribute to optimised structures and products, reducing the impact on the renewable resource, wood.

## Figures and Tables

**Table 1:** Fatigue response prediction models.

	Failure Criteria	Assumptions	Limitations
Damage accumulation models	damage variable 0<α<1	Linear damage accumulation	Not considering the load oscillation
Energy models	accumulated energy	Not considering return energy at failure	Rupture at almost any load amplitude
Number of cycles	crack size	Linear crack growth	Not considering viscoelastic effect
DVM	released energy	Initial crack	Applicable only in case of initial crack

**Table 2:** Wood fatigue influental factors.

Physical Properties/ Load	Density (LD/HD)	Moisture (LM/HM)	Temperature (LT/HT)	Size (BG/SM)	Parallel to the Grain (PAG)	Perpendicular to the Grain (PEG)	Sum	Average Value	Influ- ence
Tension (TS)	LD-TS/HD-TS	LM-TS/HM-TS	LT-TS/HT-TS	BG-TS/SM-TS	PAG-TS	PEG-TS	4.5/4.5	0.75/0.75	0.0
Compression (CS)	LD-CS/HD-CS	LM-CS/HM-CS	LT-CS/HT-CS	BG-CS/SM-CS	PAG-CS	PEG-CS	2.75/1.75	0.46/0.29	0.17
Flexure (FS)	LD-FS/HD-FS	LM-FS/HM-FS	LT-FS/HT-FS	BG-FS/SM-FS	PAG-FS	PEG-FS	3.75/3.25	0.63/0.54	0.09
Shear (SS)	LD-SS/HD-SS	LM-SS/HM-SS	LT-SS/HT-SS	BG-SS/SM-SS	PAG-SS	PEG-SS	1.75/1.25	0.29/0.21	0.08
Mean stress (LMS/HMS)	LD-LMS/LD-HMS/ HD-LMS/HD-HMS	LM-LMS/LM-HMS/ HM-LMS/HM-HMS	LT-LMS/LT-HMS/ HT-LMS/HT-HMS	BG-LMS/BG-HMS/ SM-LMS/SM-HMS	PAG-LMS/ PAG-HMS	PEG-LMS/ PEG-HMS	7/2.5	0.7/0.25	0.45
Waveform (SQ/SIN)	LD-SQ/LD-SIN/ HD-SQ/HD-SIN	LM-SQ/LM-SIN/ HM-SQ/HM-SIN	LT-SQ/LT-SIN/ HT-SQ/HT-SIN	BG-SQ/BG-SIN/ SM-SQ/SM-SIN	PAG-SQ/ PAG-SIN	PEG-SQ/ PEG-SIN	3.75/6.25	0.38/0.63	0.25
Frequency (LF/HF)	LD-LF/LD-HF/ HD-LF/HD-HF	LM-LF/LM-HF/ HM-LF/HM-HF	LT-LF/LT-HF/ HT-LF/HT-HF	BG-LF/BG-HF/ SM-LF/SM-HF	PAG-LF/ PAG-HF	PEG-LF/ PEG-HF	3.5/6.25	0.35/0.63	0.28
Sum	2.75/6.25	7.0/2.75	6.0/3.5	6.0/3.5	4.5/4.5	2.25/2.25			
Average value	0.28/0.63	0.7/0.28	0.6/0.35	0.6/0.35	0.64/0.64	0.32/0.32			
Influence	0.35	0.42	0.25	0.25	0.0	0.0			

Very unfavourable…0, unfavourable…0.25, neutral…0.5, favourable…0.75, very favourable…1. Numbers correspond to the influence of combinations of influential factors and are used to evaluate their combined effect.

**Table 3:** Effect of the combination of influential factors on wood fatigue.

Influencing Factor	Influence on Fatigue
Frequency	stronger influence with higher humidity and lower frequency.
High temperature	stronger influence on fatigue strength reduction at higher humidity.
Load outside the anatomical direction	strong influence on the reduction of fatigue strength.
Compressive load	higher fatigue strength.
High frequency, low load	low heat generation.
High load, low frequency	high heat generation.
Bending stress in tangential direction	better fatigue strength.

## Data Availability

No new data were created or analyzed in this study. Data sharing is not applicable to this article.
